# Cardiac energy metabolism and oxygenation during exercise in the hypertensive heart

**DOI:** 10.1186/1532-429X-16-S1-O109

**Published:** 2014-01-16

**Authors:** Sairia Dass, Rina Ariga, Emily Sever, Lowri E Cochlin, Joseph Suttie, Cameron Holloway, Masliza Mahmod, Theodoros D Karamitsos, Stefan Neubauer

**Affiliations:** 1 OCMR, University of Oxford, Oxford, UK; 2 Internal Medicine, St Joseph Mercy Hospital, Ann Arbor, Michigan, USA

## Background

Hypertension (HPT) is an important risk factor for heart disease worldwide. It is already established that in hypertension there is an abnormal oxygenation response to stress (by exploiting the paramagnetic properties of deoxyhemoglobin, blood oxygen level-dependent, BOLD). Additionally, abnormal resting cardiac energetics (phosphocreatine/adenosine triphosphate, PCr/ATP, as measured by 31Phosphorus MR Spectroscopy, 31P MRS) has been reported. However, it is not known if this energetic profile worsens with the increasing energy demand of exercise, and if so, if the blunted Oxygen supply response may contribute to this. Understanding this relation can lend new insights into the pathophysiology and management of hypertensive heart disease. We hypothesized that cardiac energetic are abnormal at rest and are further impaired during acute exercise in HPT, and that this impairment is related to abnormal oxygenation during stress.

## Methods

Cardiac 31P MRS (3T) was performed in 17 hypertensive patients, and 20 age and gender matched normal controls at rest and during 8 minutes of leg exercise lying prone, with 2.5 kg weights attached to both legs. BOLD (using a T2-prepared sequence) and first-pass perfusion images (using a saturation recovery fast-gradient echo sequence and 0.03 mmol/kg Gd-DTPA bolus) were also acquired at stress (4-6 minutes i.v. adenosine, 140 μg/kg/min) and rest. Signal intensity change (SIΔ) and myocardial perfusion reserve index (MPRI) were measured from BOLD and perfusion images, respectively.

## Results

**Figure 1:**
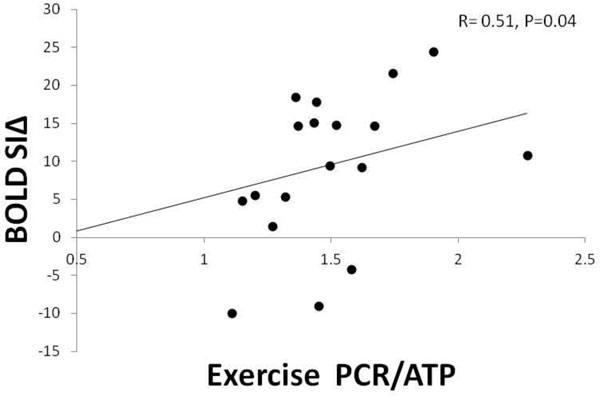
**Relationship between BOLD signal intensity change exercise cardiac energetic**.

Increases in rate pressure product with exercise (HPT 74 ± 44%, normal 73 ± 40%, P = 0.71) and adenosine stress (HPT 73 ± 46%, normal 73 ± 37%, P = 0.67) were similar. In normals, there was no change in PCr/ATP during exercise (rest: 2.16 ± 0.08, exercise: 2.15 ± 0.06, P = 0.97). Resting PCr/ATP was significantly reduced in HPT (1.63 ± 0.07, P = 0.001 vs controls), and during exercise, there was a further reduction in PCr/ATP (1.49 ± 0.07, P = 0.03 vs rest). There was a significantly reduced BOLD SIΔ response in HPT (BOLD SIΔ: 10 ± 2%; normal 20 ± 0.01% P = 0.004). MPRI was also significantly reduced in HPT (1.6 ± 0.07; normal 1.9 ± 0.02, P = 0.001). There was a weak but significant correlation between BOLD SIΔ and MPRI per segment, (R = 0.25, P = 0.006). Importantly, in HPT, there was a significant correlation between exercise PCr/ATP and BOLD SIΔ (R = 0.51, P = 0.04), Figure 1.

## Conclusions

During exercise, the pre-existing energetic deficit in HPT is further exacerbated and correlates with the blunted myocardial oxygenation response to vasodilator stress. In the hypertensive heart, myocardial tissue hypoxia during stress may play a significant pathophysiological role by inducing adverse metabolic changes in the myocardium.

## Funding

British Heart Foundation.

